# Control of the Antitumor Immune Response by Cancer Metabolism

**DOI:** 10.3390/cells8020104

**Published:** 2019-01-31

**Authors:** Charlotte Domblides, Lydia Lartigue, Benjamin Faustin

**Affiliations:** 1 Bordeaux University, CNRS, UMR 5164, ImmunoConcEpT, 33000 Bordeaux, France; charlotte.domblides@chu-bordeaux.fr; 2 Department of Medical Oncology, Hôpital Saint-André, Bordeaux University Hospital-CHU, 33000 Bordeaux, France; 3 Curematch, Inc., 6440 Lusk Bvld, San Diego, CA 92121, USA; llartigue@curematch.com; 4 Cellomet, CGFB, 146 Rue léo Saignat, F-33000 Bordeaux, France

**Keywords:** cancer, metabolism, immunity

## Abstract

The metabolic reprogramming of tumor cells and immune escape are two major hallmarks of cancer cells. The metabolic changes that occur during tumorigenesis, enabling survival and proliferation, are described for both solid and hematological malignancies. Concurrently, tumor cells have deployed mechanisms to escape immune cell recognition and destruction. Additionally, therapeutic blocking of tumor-mediated immunosuppression has proven to have an unprecedented positive impact in clinical oncology. Increased evidence suggests that cancer metabolism not only plays a crucial role in cancer signaling for sustaining tumorigenesis and survival, but also has wider implications in the regulation of antitumor immune signaling through both the release of signaling molecules and the expression of immune membrane ligands. Here, we review these molecular events to highlight the contribution of cancer cell metabolic reprogramming on the shaping of the antitumor immune response.

## 1. Introduction

Both the innate and adaptive immune systems have now established roles in the host defense against cancers through various mechanisms, which are raising an unprecedented development of modern cancer immunotherapies. Several cancers (i.e., from early neoplasia to advanced stages) are immunogenic because of their high mutational rate [1], leading to the expression of neoantigens recognized by immune lymphoid cells for further elimination. However, immunoediting (a hallmark of cancer cells) [2] is a dynamic process by which a tumor changes its immunogenicity in order to escape the immune response. It was shown to have three phases: elimination, equilibrium and escape. The elimination leads to the rejection of tumor cells by the intervention of innate and adaptive immune systems. The equilibrium state means that the immune systems have not permitted the complete elimination of the tumor cells. This is due to the genomic instability of cancer cells that can generate clones with reduced immunogenicity. During this phase, immune systems can control the tumor growth. The tumor cells enter a dormant state, sometimes for several years. These first two phases are not clinically visible, but new tumor cell variants can emerge, selecting the less immunogenic cells or cells that harbor immune resistance mutations. This leads to the final step of immune escape, where tumor cells can proliferate and overtake host immune defenses. Immune cell response depends on the expression of genetic programs, leading to the transition between quiescent, activated and memory states, and associated with profound differences in energy requirements. The immune response is associated with dramatic modifications in tissue metabolism, including the depletion of nutrients, increased oxygen consumption, and the generation of reactive nitrogen and oxygen intermediates. These modifications are, in part, due to the recruitment of many inflammatory and immune cells [3].

The most used nutrient is glucose. Glycolysis converts glucose into pyruvate in the cytoplasm. Under normoxic conditions, pyruvate is transformed into acetyl-CoA in the mitochondria, by oxidative decarboxylation. Acetyl-CoA enters the tricarboxylic acid (TCA) cycle and is oxidized into CO2, permitting the reduction of (i) nicotinamide adenine nucleotide (NAD) into NADH and/or (ii) flavin adenine dinucleotide into FADH2. These two redox cofactors can be used by the electron transport chain (ETC) for oxidative phosphorylation (OXPHOS), to create a proton gradient strong enough to permit the phosphorylation of ADP into ATP by the ATP synthase. The ETC is the most efficient way to produce energy for cell metabolism. Indeed, OXPHOS produces 36 molecules of ATP from one molecule of glucose [4]. Cells can metabolize other substrates, such as glutamine via glutaminolysis or fatty acids in β-oxidation, to replenish the TCA cycle and OXPHOS.

Cells can also produce ATP through the fermentation. Under hypoxic conditions, pyruvate remains in the cytoplasm and is converted into lactate rather than acetyl-CoA. This reaction is catalyzed by the lactate dehydrogenase (LDH). This pathway is quicker but less efficient than OXPHOS. Indeed, it produces only two molecules of ATP per molecule of glucose. Fermentation can also be done under normoxic conditions; this is the Warburg effect. Many cells, including cancer cells, prefer to use aerobic glycolysis because it is quicker and it generates precursors for the chemical constituents that form the macromolecules essential for cell division, such as nucleotides, lipids, proteins and nucleic acids [5].

## 2. Metabolism of Tumor Cells

**Figure 1:**
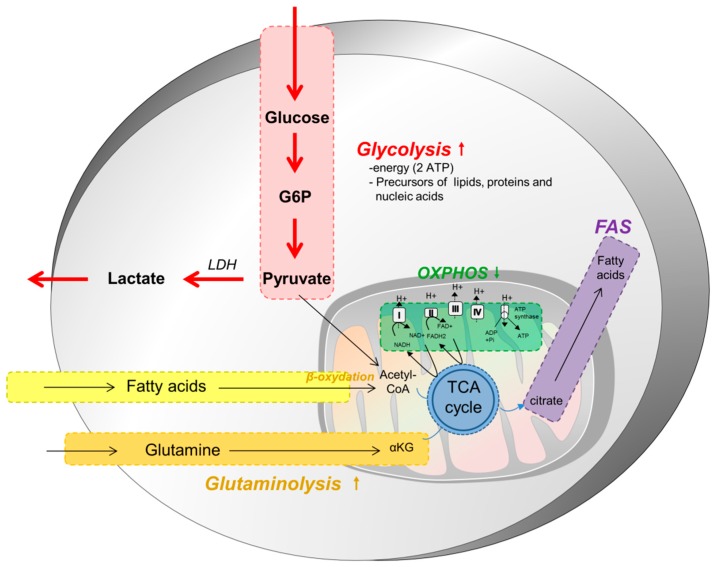
Cancer cell metabolism. Most cancer cells depend on a higher glycolytic metabolism to proliferate and disseminate, regardless of the presence of oxygen. This is made possible through the overexpression of GLUT1, a glucose cell surface transporter. This metabolic switch, known as the Warburg effect, occurs at the expense of the oxidative phosphorylation (OXPHOS) and provides, in a short amount of time, the energy, as well as many precursors of the macromolecules, required to sustain a high rate of proliferation. In cancer cells, pyruvate is largely directed to produce lactate instead of being metabolized within mitochondria. Glutamine and fatty acids constitute two other substrates used preferentially by tumor cells, and which provide intermediates of the tricarboxylic acid cycle (TCA) cycle. They are further used to produce other building blocks for these demanding cells and to maintain some mitochondrial activity and functioning.

Tumor cells modify their metabolism in order to supply energy for cell growth and proliferation. Metabolic reprogramming is now an established hallmark of cancer [2] and is targeted in the clinic. In most solid tumors, cancer cell metabolism is characterized by the Warburg effect, which means that, even in the presence of oxygen and fully functioning mitochondria, tumor cells preferentially use aerobic glycolysis and lactate fermentation as opposed to oxidative phosphorylation (Figure 1). This metabolic reprogramming permits the production of energy, but also nutrients as lipids, proteins or nucleic acids, necessary for the generation of daughter cells. This reprogramming leads to several modifications in nutrient uptake, the activation of metabolic pathways and in interactions with the microenvironment, modifying the fate of tumor cells and components of the microenvironment.

Because aerobic glycolysis is less efficient for the production of ATP, tumor cells increase their nutrient uptake from the microenvironment [6]. The two main nutrients used by cancer cells are glucose and glutamine (Figure 1). These nutrients permit the maintenance of the pool of carbon intermediates in the cells, to generate NADH and FADH_2_ for ATP production, NADPH for biosynthetic reactions or to maintain redox balance in cells. Aerobic glycolysis has low efficiency and cancer cells upregulate transporters in order to increase glucose uptake, notably that of GLUT1 [7]. This increased glucose uptake is normally regulated by extracellular growth signals and by the interactions between cells and the matrix; however, cancer cells become insensitive to these external signals due to numerous genomic alterations (activation of Pi3k/Akt/mTOR pathway or Ras) [7]. The Pi3K pathway plays a central role in this metabolic reprogramming as a master regulator of glucose uptake and metabolism. This pathway is induced by hypoxic conditions, through the production of Hypoxia-inducible factor 1-alpha (HIF-1α). mTOR upregulates the translocation of GLUT1 at the cell surface [8,9]. Furthermore, hypoxia conditions also induce the overexpression of several glycolytic enzymes such as hexokinase and phosphofructokinase activities, through Akt activation [10]. Cancer cells also inhibit the mitochondrial TCA cycle through the upregulation of pyruvate dehydrogenase kinase (PDK) expression and mutations of fumarate hydratase (FH) and succinate dehydrogenase (SDH), while promoting glycolysis.

Glutamine also plays an important role in cancer cell metabolism and proliferation. It is involved in cell proliferation through its transformation into α-ketoglutarate, which enters the TCA cycle for the production of amino acids, nucleotides and fatty acids [5,11]. Furthermore, glutamine is also important for the production of glutathione, involved in redox homeostasis. C-Myc is the principal driver of glutamine metabolism [12], which increases the expression of glutamine transporters and their metabolic enzymes [13]. Furthermore, the loss of the retinoblastoma (Rb) tumor suppressor is also involved in increased glutamine uptake and metabolism as it blocks the uptake of glutamine [14,15]. It is worthwhile to note that glucose and glutamine are also two metabolites required for the activation of naive T cells [16,17]. Upon stimulation, T cells undergo a Warburg-like metabolic reprogramming, enhancing glucose uptake and glycolysis to promote proliferation [18]. They also require glutamine to generate various intermediates as well as precursors of protein and lipid biosynthesis to complete differentiation and fulfill their tasks [19]. These common requirements create a metabolic competition within the tumor microenvironment (TME), where oncogene-driven cancer cells eventually take control and deplete surrounding nutrients upon proliferation, at the expense of the immune cell’s fitness and survival, thereby blunting immune responses [19].

In addition to glucose and glutamine addiction, cancer cells also rely on the so-called ‘one-carbon metabolism’ to sustain their high rate of proliferation [20]. One-carbon units are indeed indispensable for nucleotide production, methylation processes and NADH/NADPH pool renewal. The serine-to-glycine pathway constitutes one major source of one-carbon units and, as such, is often diverted and over-activated in tumors to favor their growth. Cancer cells generally increase their serine supply, by up-regulating the intracellular de novo serine synthesis pathway or by augmenting its uptake from the local environment [21,22,23]. This specific metabolic dependency is of therapeutic interest as serine deprivation has been shown to inhibit cancer cell proliferation. However, recent advances have also revealed the importance of serine in T cell expansion upon activation [24], thereby demonstrating a metabolic competition between tumor and immune cells for this important amino acid. It is therefore crucial to further improve knowledge of this pathway to target tumors without impairing T cell function and immune response.

Finally, cancer cells also undergo a modified lipid metabolism that is mostly characterized to a lipogenic phenotype. Indeed, they overexpress enzymes involved in the synthesis of saturated lipids.

As previously described, profound metabolic changes occur within immune cells upon activation as part of their response to invading pathogens or injury [3]. Lymphoid cells generally switch from mitochondrial OXPHOS to aerobic glycolysis to become fully active and proliferate when they encounter a danger signal. As soon as the clearing is finished, the remaining memory cells undergo a reversed metabolic switch, correlating to their reacquisition of a quiescent non-proliferative state. Dendritic cells also rely on higher glycolytic flux to be activated, as do proinflammatory macrophages upon M1 polarization. As for anti-inflammatory M2 polarized macrophages, they are mostly dependent on mitochondrial OXPHOS and fatty acid oxidation (FAO). All these metabolic changes are key to an efficient immune response in normal or disease conditions, including cancer, as detailed in [3]. Although further details on the immune cells metabolic requirements won’t be discussed here, it is important to consider that immune cells are permanently competing with tumor cells for nutrient resources (glucose, glutamine, serine, tryptophan) in order to proliferate and function properly [16,25]. Metabolic competition is therefore poised as one crucial step in the immune hypo-responsiveness observed in cancer [26].

Aside from immune cells, the whole tumor niche influences the metabolic status of cancer cells and vice versa [27]. Intricate relationships exist between a cancer cell and its microenvironment, usually including non-tumor cells such as fibroblasts, adipocytes, endothelial cells and immune cells, as well as multiple soluble secreted factors. This complex network guides and shapes the metabolic balance of the tumor and, in turn, its behavior. Although the global metabolic cooperation/competition between the various components of the TME are not the point of this review, it is worth mentioning that, ultimately, these cells are organized to promote cancer growth and facilitate dissemination [27]. Indeed, as mentioned, competition between cancer cells and T cells for certain metabolites leads to immune suppression, while also supporting the polarization of pro-tumorigenic M2 macrophages that rely on OXPHOS and FAO [16]. Highly glycolytic cancer-associated fibroblasts (CAF) build up a favorable milieu for cancer cell development, releasing lactate, acidifying the milieu and providing high energy metabolites intermediates to fuel proliferation. Additionally, adipocytes turn into cancer-associated adipocytes within the TME, offering a vital source of fatty acids for the quickly dividing and energy demanding tumor cells [28].

## 3. Effect of Cancer Metabolism on Infiltrating Immune Cells

Immune escape is a major hallmark of cancer cells [2]. Tumor cells disable immune components that are activated to eliminate them. Several mechanisms can lead to this escape: reduced expression of antigens at the surface of tumor cells, reduced expression of major histocompatibility (MHC) molecules by antigen-presenting cells (APC), impaired co-localization of T-cell receptor (TCR) and co-stimulatory receptors, secretion of inhibitory cytokines and activation of inhibitory receptors on T cell surface. This leads to a selection of tumor cells that are resistant to the immune response and that generate an immunosuppressive and pro-tumoral microenvironment. Immune cells acquire functional defects and enter a hyporesponsive (or anergic) reversible state, with impaired effector capacities favoring tumor progression. Next, we discuss how cancer metabolism triggers immune escape mechanisms and we review the implications of this in current immunotherapy strategies.

### 3.1. Medium Acidification and Lactate Accumulation

#### 3.1.1. The Production of Lactate by Tumor Cells

As discussed above, tumor cells derive their energy from a number of nutrients, mainly glucose and glutamine. Both pathways produce significant amounts of lactate because of the overexpression of the lactate dehydrogenase (LDH) and the greater rate of glucose consumption. Lactic acid accumulates in the cytoplasm and extracellular medium because of high rates of production and insufficient vascular clearing [29]. During this process, acidification can occur, based on the co-transport of protons with lactate export, through the Mono-Carboxylate Transporter 4 (MCT4). MCT4 controls intracellular pH. It has a low affinity for lactic acid and a high capacity for transport in order to meet the high rates of production and utilization of lactate [30,31]. Tumor cells release lactic acid in the extracellular medium since intracellular pH variation can alter cell survival and metabolism, motility, apoptosis, and biosynthesis [32,33].

Furthermore, tumor cells overexpress membrane Carbonic Anhydrase IX (CA-IX), contributing to further acidification of the tumor microenvironment [34]. This enzyme catalyzes the transformation of carbon dioxide into bicarbonate and protons. Its expression is controlled by the HIF pathway and is associated with poor prognosis and a higher rate of metastasis [35]. This enzyme preserves intracellular pH and ATP levels in vitro and in vivo, leading to the promotion of cell survival and growth [34]. Tumor cells are able to control the extracellular pH to promote their proliferation and survival. They modulate their own metabolism to regulate lactate efflux [36]. Indeed, Warburg metabolism seems to be a dynamic negative feedback loop permitting the regulation of the microenvironment pH by actively modifying the balance between OXPHOS and glycolysis. Under extracellular acidification, tumor cells can attenuate glucose consumption, reduce lactate production and increase OXPHOS gene activation in order to limit further acidification. Therefore, tumor cells can control their microenvironment and maintain an aggressive phenotype.

Lactic acid is involved in the tumor phenotype. First, high levels of lactate can induce the degradation of the extracellular milieu, promoting migration and more frequent metastasis in a dose-dependent manner in vitro [37,38]. The knockdown of LDH inhibits the migration of glioma cells in vitro and this effect is reversed by supplying lactic acid in the medium [39]. The acidification of the extracellular environment also modifies tumor cell migration. Pharmacological or genetic inhibition of sodium/proton antiport was shown, for instance, to reduce the migration potential of hepatocarcinoma cell lines (without effecting their proliferation), through a reduced expression of metalloproteinase MMP-2 [40]. Furthermore, lactic acid induces the expression of protumor molecules, such as hyaluronic acid from tumor-associated fibroblasts and TGF-β [41,42], that are also involved in invasive phenotypes. Indeed, in vitro melanoma models showed that tumor cells overexpressed CD44 under acidic conditions (through lactate response elements in CD44-promoter) to retain hyaluronic acid on their surface and to favor their aggressive phenotype [29].

In addition, lactic acid has an angiogenic role, through the coupling between lactate and pyruvate via LDH, by inducing the expression of HIF-target genes [43]. The acidification leads to the elevation of IL-8 (endothelial cells) and VEGF (macrophages and endothelial cells) amounts, which are both proangiogenic factors involved in the metastatic process [44]. IL-8 inhibition through RNA interference leads to the inhibition of angiogenesis in tumors [45]. Finally, acidification increases tumor cell survival [46], and is associated with radioresistance in vitro and in vivo [47], possibly because of its antioxidant properties [48], which protect cancer cells from damage caused by radiation.

#### 3.1.2. Role of Lactate and Extracellular Medium Acidification on Immune Cells

**Figure 2:**
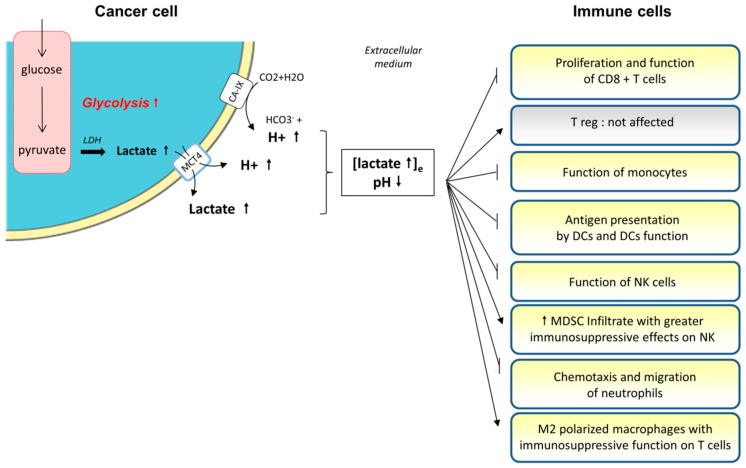
Cancer cells induced-lactate and -proton release within the peritumoral environment and the consequences for immune cell function. The metabolic switch towards aerobic glycolysis observed in most cancer cells (i.e., Warburg’s effect) produces significant amounts of pyruvate that are mostly metabolized into lactate. The intracellular increase of lactate, potentially toxic for the cancer cell, is then released into the extracellular medium together with protons (thanks to transporters such as MCT4), inducing an acidification of the local environment. The frequent up-regulation of carbonic anhydrase IX (CA-IX) in cancer cells also contributes to the local increase of H+ in the immediate neighborhood of cancer cells. Both, the accumulation of lactate and the decrease in the pH of the extracellular milieu create a detrimental environment for the antitumoral immune response, (i) decreasing the function of the CD8+ T cells, dendritic cells (DCs), monocytes, natural killer (NK) cells and/or (ii) increasing the myeloid-derived suppressor cells (MDSC) infiltrate or M2-polarized macrophages with greater immunosuppressive functions on NKs and T cells, respectively.

The effects of acidosis on immune functions are associated with immunodeficiency in non-cancer diseases such as sepsis [49]. In cancer, acidification also provides a growth advantage to tumor cells at the expense of immune cells [38,50,51] (Figure 2). The tumor releases lactic acid in order to acidify the medium, leading to higher concentrations and a lower pH in the extracellular medium. This pH gradient induces the blockade of lactate efflux from T cells and its accumulation in the T cell’s intracellular medium [50]. Medium acidification leads to CD8^+^ TILs (Tumor Infiltrating Lymphocytes) anergy in human and mouse models [50,52]. The accumulation of lactic acid induces a diminution of T cell proliferation, after antigen stimulation, in a dose-dependent and time-dependent manner [50]. This inhibition occurs immediately after the contact of T cells with lactate. Acidification also impairs their function (reduced cytokine secretion and cytolytic activity through reduced expression of granzyme B) and decreases their activation (reduced TCR expression and STAT5 and ERK activations) [52]. Furthermore, prolonged exposure (at least 24 h) to lactic acid induces apoptosis of more than 60% of T cells, whereas a shorter exposure leads to only a diminished function of T cells without impairment of their survival. These effects were not found when the acidification of the medium was done with hydrochloric acid, meaning that lactic acid is toxic to T cells with a mechanism other than the sole acidification of the medium. This anergy is a reversible state, because treatment of mice with a proton-pump inhibitor (PPI) restored their immune effector function and increased the efficacy of adoptive immunotherapy [52]. These effects of lactate on T cells are possibly due to the impairment of the TCR-triggered activation of c-Jun N-terminal kinase (JNK), c-Jun and p38 [53]. Mitogen-activated protein kinase (MAPK) and mTOR pathways are not affected. In contrast, regulatory T cells (Treg) do not seem to be impaired by lactate and acidification, because of a different metabolism, based on fatty acid oxidation (FAO) [54].

Lactate accumulation in the extracellular medium also alters monocyte function and metabolism. Indeed, it impairs the tumor necrosis factor (TNF) secretion of monocytes in vitro without effecting their viability [55]. This effect is due to an increased uptake of lactate and the co-transport of protons through MCT-1 and MCT-4, because of the pH gradient. There is also an inhibition of reactive oxygen species (ROS) production, which is important to the cytotoxic activity. All these effects were reversible after the administration of the lactate inhibitor oxamic acid. Lactic acid reduces the expression of genes involved in the inflammatory effector functions of monocytes, such as in the expression of cytokines (IL-23 and TNF mostly) and chemokines (CCL2 and CCL7). It also delays the phosphorylation and activation of Akt (involved in glycolysis), and reduces nuclear-factor (NF)-κB accumulation in monocytes (which regulates the expression of cytokines and chemokines) [56]. Extracellular lactate was shown to be sufficient to switch inflammatory M1 macrophage polarization towards immunosuppressive M2; the latter being evidenced to accumulate in the tumor microenvironment [57].

Lactic acid does not alter monocyte differentiation into dendritic cells (DCs) in the in vitro models. However, lactate-mediated acidification induces an alteration of antigen presentation by DCs, and of their functional activity. This leads to the acquisition of tumor-associated DC phenotypes, and the blockade of lactate restores their normal phenotype [58]. These tumor-associated DCs have a particular pattern for the presentation of antigens, which favor tumor progression. These effects are probably due to the secretion, by tumor-favoring DCs and tumor cells, of IL-6 and growth factors such as macrophage colony-stimulating factor (M-CSF). Furthermore, lactate increased the production of IL-23 in human lung adenocarcinoma cell lines [59]. IL-23 is overexpressed by macrophages and DCs in tumors and promotes tumor growth [60]. It is an important molecule that upregulates IL-17, IL-6 and TNF-α secretion, with an autocrine and paracrine effect. These cytokines have a proinflammatory effect, without increasing immune infiltration, because of reduced Th1-dependent interferon (IFN)-γ secretion. Furthermore, IL-23 increases the expression of the matrix metalloprotease 9, the angiogenesis, and reduces CD8^+^ T cell infiltration in the tumor microenvironment [59]. Indeed, models of a tumor-xenograft mouse which harbor a deletion of IL-23 have restricted tumor growth [60].

Lactic acid also acts on other immune cells. Indeed, through its induction of HIF expression, it induces the expression of arginase I and iNOS in M2-polarized macrophages [61], harboring an immunosuppressive function on T cells. On natural killer (NK) cells and myeloid-derived suppressor cells (MDSC), mouse models have shown that lactic acid decreases the function of NK cells, through a diminution of the expression of granzyme B and perforin and a diminution of the expression of the activating receptor NKp46 (which plays an important role in their cytotoxicity) [62]. Lactic acid also enhances the infiltration of MDSCs in a peritumoral environment, with greater suppressive effects on NK cells. Finally, lactic acid leads to the inhibition of chemotaxis and the migration of polymorphonuclear neutrophils [63,64,65].

#### 3.1.3. Impact in Clinical Routines

High levels of lactate in primary tumors are correlated with a poor outcome and an increased rate of distant metastasis in several cancers [66,67,68]. Targeting lactate production and its repercussions remains, therefore, attractive through various therapeutic strategies, as described below.

This can be done through alkalization of the medium. PPI, used in gastro-intestinal diseases, inhibits the vacuolar-ATPase, whose expression is enhanced in several tumors. This ATPase is a proton pump involved in medium acidification. Its inhibition decreases proton extrusion from cells and leads to extracellular pH alkalization. It induces cytotoxicity in melanoma cells in vitro, through the acidification of the intracellular medium (pH gradient), leading to the activation of caspases and the apoptosis of tumor cells, independently of the mutational profile [29]. In mouse models, PPI permitted reduced tumor growth and increased survival in melanoma-bearing animals. The extracellular alkalization can also favor the immune cell efflux of lactic acid, and restore their effector functions. The administration of PPI is safe because its activation depends on a medium pH. Indeed, PPI is only activated under acidic conditions, targeting its activation mainly in tumor tissues.

One Phase 2 clinical trial has been completed (NCT01069081), assessing the efficacy and safety profile of concomitant administration of docetaxel and cisplatin chemotherapy with high doses of PPI in metastatic breast cancer. Results showed an improvement of the antitumor effect of the chemotherapies, inciting a Phase 3 study [69]. Another way to inhibit acidification and restore immune response is the use of sodium bicarbonate [70]. Thus, treatment of tumor-bearing mice with oral sodium bicarbonate lead to an increase in the pH and to lower metastasis, but not in all tumor models. Preliminary investigations showed that alkalization reduced the release of cathepsin B in the extracellular medium, an important matrix remodeling protease.

Next was the inhibition of lactate production. Oxamate is a pyruvate analogue that competitively inhibits LDH isoform A (LDH-A). In a xenograft mouse model, it was reported that the inhibition of LDH-A activity leads to the inhibition of aerobic glycolysis in tumor cells, whereas normal cells were not impaired [71]. In vitro, oxamate was shown to have a proapoptotic effect via the activation of caspase 3 and the expression of Bax [72]. It suppresses the proliferation of lung cancer cell lines, with a lower effect on normal cells [73]. This effect was due to a reduction of ATP and ROS and to a phase G0/G1 arrest. Others strategies for inhibiting LDH-A, such as siRNA, have been assessed [74]. No ongoing trials are currently assessing these molecules in cancer.

### 3.2. Indoleamine 2,3-Dioxygenase (IDO) and Tryptophan Dioxygenase (TDO)

#### 3.2.1. IDO Characteristics

The tryptophan dioxygenase (TDO) was first described as the only enzyme capable of metabolizing L-tryptophan, an essential amino acid. Later, however, indoleamine 2,3-dioxygenase (IDO) was discovered in rabbits and shown to be able to metabolize both l- and d-tryptophan [75]. There are three isoforms existing, IDO1, IDO2, and TDO2. These enzymes have different inducers as well as different tissue expression patterns [76]. Indeed, IDO1 is induced by proinflammatory cytokines such as IFN-γ, LPS and pathogens like influenza viruses [77,78]. This is widely expressed across most organs, in plasmacytoid DCs of lymph nodes and the spleen, macrophages, endothelial cells, stromal mesenchymal cells and fibroblasts [79,80]. TDO2 is induced by tryptophan itself and glucocorticoids, and is expressed in the liver, brain and placenta, and IDO2 is expressed in hepatocytes, neuronal cells and DCs [81].

IDO is encoded by the *IDO1* and *IDO2* genes in human chromosome 8p11 and *TDO2* gene in chromosome 4q32. It was the first IFN-activated gene identified in the 1970s [82]. It is a cytosolic enzyme which catalyzes the first step of the tryptophan catabolism in the kynurenine pathway (catabolism of tryptophan into N-formyl-kynurenine). Tryptophan metabolism is important for the production of the energy cofactor NAD^+^. The enzyme is a 407 amino acid heme-containing protein. In mice, IDO was described as a protein that prevents fetal rejection [83]. In humans, IDO modulates antigen-dependent activation of immune cells on the mucosal surfaces of lungs and the digestive intestine [80]. Furthermore, it prevents excessive cytotoxic immune response leading to tissue damage.

The *IDO1* promoter contains ISREs (IFN-stimulated response elements) and GASs (IFNγ-activated sites). Several transcription factors can translocate into the nucleus in order to enhance the expression of IDO1. IFN-γ is the most potent IDO1 inducer. Similar to LPS, it activates the Janus kinase/signal transducer and activator of transcription (JAK/STAT) pathway, which leads to the expression of STAT1 or STAT3 [84]. Kynurenine, its metabolite, through its interaction with the aryl hydrocarbon receptor (AhR), can also induce IDO1 expression through the STAT3 pathway. Others transcription factors can also activate IDO1 transcription: IRF1 (IFN regulator 1) [85], the NF-κB pathway and ETV4 (ETS variant 4) [84].

#### 3.2.2. IDO Expression in Tumor Cells

IDO is associated with numerous immune diseases, as diverse as cancer, allergies, autoimmune and inflammatory diseases. IDO1 can have two expression patterns. In some tumors, IDO1-expressing tumor cells are in lymphocyte-rich areas, meaning that IDO-expression can be the consequence of IFN-γ expression and a resistance mechanism. In other cancers, IDO1 expression is constitutive and IDO1 expressing tumor cells are surrounded by less lymphocytes. In vitro, several cell lines can constitutively overexpress IDO, despite the absence of IFN-γ, with variable levels of activity according to cell lines [86,87]. This is explained by Bin1 mutations [88]. Bin1 is a tumor suppressor gene encoding an adaptor protein, the Bin1/amphiphysin/Rvs167 (*BAR*). It is found to be attenuated in several cancers, promoting proliferation, motility and survival [79]. In vivo studies have shown that the main consequence of Bin1 inactivation is the increase of intracellular amounts of STAT1 and NF-κB, leading to the upregulation of IDO expression. Its expression was also found in peritumoral cells, but not in distant stroma.

IDO activity can also be induced by several factors, such as the oncogene Kit that is commonly altered in several cancers. Once activated, Kit induces ETS variant 4 (ETV4) in cytoplasm. Furthermore, IDO1 can sustain its own expression through an autocrine loop [89]. Indeed, the IDO1 gene can be activated by the binding of kynurenine-AhR on its response elements, activating STAT3. STAT3 can induce expression of IDO1 and IL-6, which exerts an autocrine/paracrine feedback loop based on the interaction between IL-6 and its receptor that enhances expression of STAT3.

IDO acts at multiple levels of tumorigenesis, all associated with inflammation: metastatic process, immune escape, invasion and angiogenesis [79]. IDO seems to be an integral component of chronic inflammation, required to support tumor development in chronic inflammatory models [90]. There is probably an interconnection between inflammation and immune escape programs, because IDO is expressed only until some degree of inflammation occurs in the tumors [87]. IDO acts at different stages by favoring tumor progression and metastatic evolution [79], by maintaining a proinflammatory and protumor microenvironment. Indeed, IDO1 deficient mice are resistant to tumorigenesis [91], develop less lung metastasis, have a lower IL-6 amount and have better survival rates [92]. Furthermore, these deficient mice have impaired angiogenesis in the lungs, even in the absence of cancer. IDO seems to display a more complicated role, beyond its immunomodulatory, pro-metastatic and angiogenic functions.

**Figure 3:**
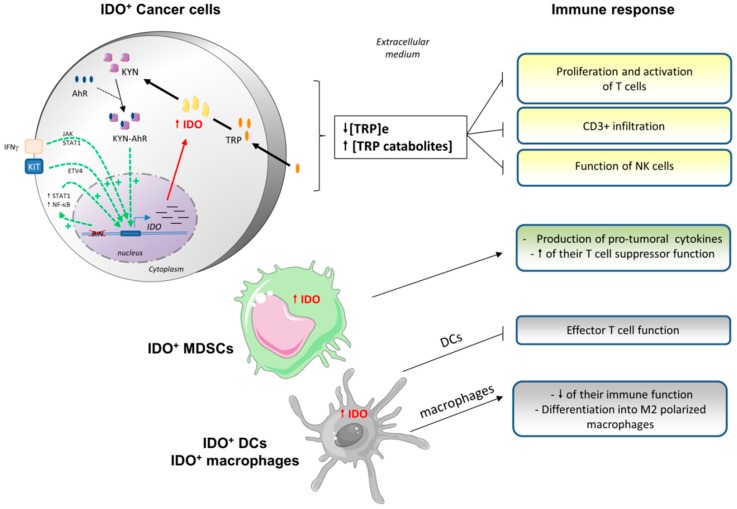
Consequences of indoleamine 2,3 dioxygenase (IDO) overexpression on the antitumoral immune response. Cancer cells frequently overexpress IDO as a result of its transcriptional upregulation. This can be triggered by (i) cytokines such as IFNγ, as long as lymphocytes are present within the tumor, (ii) the oncogene Kit, or (iii) mutations within the tumor suppressor gene Bin. IFNγ promotes IDO overexpression by activating the Janus kinase/signal transducer and activator of transcription (JAK/STAT) pathway, while Kit and Bin stimulate ETV4 (ETS variant 4), and STAT1 and/or nuclear factor (NF)-κB, respectively. IDO expression levels can also be regulated by an autocrine loop, implying the complex KYN-AhR. Once in the cytosol, IDO mediates the transformation of tryptophan (TRP) into N-formylkynurenine that is then transformed into kynurenine (KYN). IDO overexpression leads to a decrease in the extracellular level of TRP, which limits the action of immune cells: T cells are not proliferating and can’t be activated; and CD3+ cells infiltrate less frequently and the function of NK cells is reduced. Cells other than cancer cells can overexpress IDO, including some immune cells such as MDSCs and DCs/macrophages. The overexpression of IDO in such cells restrains their own immune function (macrophages), suppresses T cells activity (DCs), and induces protumoral cytokines (MDSCs). As a result, in IDO overexpressing tumors, the antitumoral immune response is largely compromised.

IDO can be overexpressed either in tumor cells or in tumor-associated cells such as dendritic cells, macrophages, or endothelial cells (Figure 3). Indeed, its overexpression leads to tryptophan deprivation, which can impair the immune cell functions, creating a de novo local tolerance, due to the anti-inflammatory effect [93]. IDO1 overexpression in tumor cells also leads to reduced plasmatic amounts of tryptophan, meaning that the immunosuppressive effect of IDO1 is not locally restricted but is systemic [94].

Next we review the effects of tryptophan metabolism on lymphocytes. The overexpression of IDO by tumor cells leads to a tryptophan deprivation in the extracellular medium (Figure 3). T cells are very sensitive to the variation of tryptophan abundance in their microenvironment. Thus, this deprivation has effects as soon as the level decreases lower than 0,5–1 µM [95,96]. This deprivation leads to anergy and to a reduced proliferation in vitro and in vivo [97].

IDO overexpression can block TILs proliferation through two mechanisms: by direct tryptophan deprivation in the microenvironment (GCN2 activation and mTOR inhibition), and by indirectly induced toxicity of tryptophan catabolites [86,98]. The accumulation of uncharged tryptophan-tRNA, due to tryptophan deprivation, induces the enhancement of stress signals, mediated by GCN2 (general control nonderepressible 2) which phosphorylates the transcription factor EIF2α [99]. GCN2 activation leads to the polarization of CD4^+^ T cells into Tregs cells from Th17 [99,100]. Tryptophan deprivation also induces an impairment of their antigen-dependent activation [101], downregulation of the TCR ζ-chain [100], suppression of factors needed for the downstream signaling of the TCR through the MAPK pathway [102], and activation of autophagy through the inhibition of mammalian target of rapamycin (mTORC1) and protein kinase C (PKC-Θ) [103]. IDO expression also leads to the lower infiltration of CD3^+^ cells [104]. Finally, tryptophan deprivation increases the sensibility of T cells to Fas-mediated apoptosis [105], but this proapoptotic effect is still controversial [86]. Several studies have shown that its supply restores T cell function.

Furthermore, tryptophan catabolism produces soluble metabolites directly toxic to several immune cells. Kynurenine is one of these immunosuppressive metabolites [106,107], probably by its interaction with aryl hydrocarbon receptors (AhR) [108]. AhR has a role in the regulation of immune response, inflammation and carcinogenesis. After its binding to AhR, kynurenine-AhR translocates to the nucleus and induces proinflammatory gene expression. The binding between kynurenine and AhR favors pathologic inflammation in the microenvironment, which has a protumoral role and facilitates escape to immune surveillance. Kynurenine-AhR has an effect on CD8^+^ TILs and CD4^+^ T_H_1 cells, through the induction of caspase 8 and cytochrome c [109,110], but not on T_H_2 cells [111]. Other catabolites, such as 3-hydroxyanthranilic acid (3HAA), also block T cell activation, inducing apoptosis through the depletion of intracellular glutathione and the inhibition of NF-κB activation [105,112]. Catabolites seem to have the same effects on B cells and NK cells in vitro [113]. Indeed, in NK cells, IDO induces the impairment of their cell-mediated killing function by the down-regulation of the activation of several receptors such as NKG2D and NKp46, without effect on the other receptors [114].

Resting Tregs must be activated to become immunosuppressive T cells. When tolerogenic IDO-expressing DCs activate resting Tregs through the TCR, the activation of the Akt pathway can favor its phenotype into helper T cells [93]. Tregs inhibit these signals via different mechanisms in order to preserve the immunosuppressive phenotype. First, they maintain durable Akt inhibition, which is a key element for Treg activation [115,116]. Indeed, they inhibit mTOR activation through amino acid deprivation, GCN2 activation, and upregulation of PTEN, thanks to the expression of PD1 and neuropilin-1. Catabolites of tryptophan also promote the expansion of some T cells into Tregs, depending on TGF-β and FoxP3 expression [100,117]. There is a positive feedback loop between DCs and Tregs. Indeed, IDO-expressing tolerogenic DCs induce Treg differentiation. In turn, Tregs express CTLA-4 that enhance IDO-expression in tolerogenic DCs [118,119]. As a consequence, in clinical practice, CTLA-4 blocking can significantly inhibit IDO expression [120].

Here we discuss the effects of tryptophan metabolism on DCs and macrophages. IDO-expressing DCs induce a tolerogenic phenotype on naive DCs and favor the IDO-expression. The binding of kynurenine on AhR [85] enables them to suppress effector T cell responses [121,122]. Indeed, this interaction inhibits the production of IL-12 and favors the secretion of IL-10 and TGF-β, leading to the modification of their antigen-presenting ability and favoring a tolerogenic phenotype [90,123,124]. The inhibition of IDO with 1-MT or siRNA in DCs leads to the diminution of the expression of costimulatory molecules [125]. It is also involved in their maturation and migration in vitro [125].

IDO expression in DCs is induced through several pathways. First, DC-secreted TGF-β activates the non-canonical NF-κB pathway and the phosphorylation of IKKα. This leads to the enhancement of IDO expression and, also, to TGF-β, with an autocrine/paracrine effect on APC. Then, IL-6 secreted by tumor or immune cells induces expressions of IDO1, such as IL-1β. PGE2 or TNF-α also induces PKA which favors IDO expression. IDO expression can also be induced by negative coregulatory membrane molecules such as CTLA-4, CD200, and GITR through IRF-1 or STAT1 [118,119,126,127], thereby amplifying antigenic tolerance. In particular, CTLA-4 induces an IFNγ-dependent expression of IDO [119].

In macrophages, one tryptophan metabolite, 3-HAA, suppresses NO secretion due to the inhibition of NF-κB and iNOS [81]. Because NO is crucial for its ability to eliminate tumor cells, 3HAA has an immunosuppressive effect on macrophages. Cells expressing IDO can co-express iNOS in response to IFN-γ, which produces NO that inhibits, in turn, IDO. Thus, several cells can induce IDO and factors that also inhibit IDO activity, in order to regulate its expression. Furthermore, IDO favors their differentiation into the M2 phenotype [128]. Tryptophan deprivation also plays an important role because it regulates cytokine secretion and favors inflammation [129].

Finally, here we discuss the effects of tryptophan metabolism on MDSCs. Some studies have found that high levels of IDO expression in MDSCs and IDO-expressing MDSCs highly inhibit the antitumor immune response [130]. IDO1 induces the expression of pro-tumoral cytokines such as IL-6 [92], which recruits MDSCs at the tumor site. As discussed above, IL-6 induces the expression of IDO1 by MDSCs through an autocrine loop [89]. The deficit of IL-6 leads to a diminution of the generation and the function of MDSCs, a diminution of their T cell suppressor function, leading to a diminution of tumor progression and to its metastatic potent. These IL-6 amounts were associated, in patients, with more frequent relapses [131]. Furthermore, local IDO recruits and activates MDSC through IDO-activated Tregs [132].

In conclusion, IDO is upregulated in tumor cells, either constitutively or upon IFN-γ response. This leads to a deprivation of tryptophan in the microenvironment, and the release of toxic catabolites. Both induce an immunosuppression on DCs and cytotoxic T cells that become incompetent for an efficient antitumor immune response [91]. Furthermore, IDO induces a tolerogenic phenotype on APCs and the generation of immunosuppressive cells such as Tregs and MDSCs. This immunosuppression can be local as well as systemic due to plasmatic tryptophan deprivation.

#### 3.2.3. Clinical Targeting of IDO

IDO overexpression and elevated amounts of AhR in tumors have a negative prognosis in several cancers (ovarian, leukemia, colorectal, cervical and endometrial) [80,86,104,133]. Furthermore, high IDO1 expression is associated with more frequent metastasis [104]. IDO targeting is very interesting because several tumors overexpress this enzyme, even in the absence of IFN-γ stimulation. Three strategies can be developed to limit IDO effects: blockade of its expression (inhibition of NF-κB, Jak/STAT or Bin1), blockade of its activity with enzymatic inhibitors, or blockade of its downstream signaling (indoximod).

First, we present the indoximod or 1-Methyl-d-tryptophan strategy. The 1-Methyl-D-tryptophan (1-MT) is a competitive inhibitor and a tryptophan analogue [134]. Indoximod suppresses the downstream effects of IDO activation, through the mTOR pathway [103]. The treatment of IDO^+^ mice with 1-MT leads to more efficient tumor rejection than that of non-treated mice [86]. The 1-MT is well tolerated in mice models, because it exerts no inhibition on TDO [86]. Thus, TDO is a liver enzyme that regulates plasmatic levels of tryptophan, avoiding tryptophan deprivation in non-tumor cells [135]. One of the expected side effects is the auto-immune phenomenon and the activation of latent diseases by the inhibition of the negative feedback on immune cells.

A Phase 1 clinical study (NCT00567931) was recently published assessing indoximod in 48 patients with metastatic solid malignancies [136]. The response rates were modest, with only five stabilizations, but a good safety profile was observed. Further investigations are needed to optimize the biomarkers selection of patients that could benefit from indoximod. Furthermore, the modest activity of this single-agent can be explained by its cytostatic activity. The next trials must assess combinations of indoximod with others antitumor therapies. Indeed, it could drastically increase the efficiency of chemotherapies [88], as shown in the murine models of spontaneous breast tumors. Furthermore, in glioma mouse models, concomitant administration of temozolomide and indoximod led to a significant decrease in tumor proliferation [137]. In an ex vivo model of breast cancer, its association with paclitaxel led to reduced tumor growth and IDO expression [138]. This efficacy is not associated with increased bioavailability of the molecules, but an immunomodulatory effect via the relief of the T cell blockade.

Furthermore, in mouse glioma models, the association of inhibition of IDO, CTLA-4 and PD-L1 led to the enhancement of the long-term survival of 100% of the mice and to a decrease of Treg in the tumors [139,140]. The immunosuppression of T cells before 1-MT treatment reduced its efficacy, meaning that its effect is probably dependent on the T lymphocyte response. Indeed, the indoximod is actually evaluated in association with adoptive cell therapy, immunotherapies like ipilimumab, or chemotherapies, in several kind of cancers (lung, prostate, breast, pancreas, brain tumors), with Phases 1 and 2 currently recruiting (see clinicaltrials.gov).

Other enzymatic inhibitors include two competitive inhibitors of IDO, INCB024360 and NLG919, which were developed in order for more specific crystallography assessment. INCB024360 (Epacadostat) is a hydroxyamidine compound which selectively inhibits IDO1, diminishing plasmatic kynurenine in mouse and dog models [141]. A Phase ½, in combination with pembrolizumab (anti-PD1), showed a positive response rate (57%) and disease control rate (86%), with a suggestive safety profile in 19 advanced cancer patients [142]. It is actually assessed in several trials in association with chemotherapies or others immunotherapies.

IDO is spontaneously recognized by CD8^+^ T cells, leading to the activation of cytotoxic activity [143,144]. Thus, small parts of IDO peptides can be used for vaccinotherapy. A Phase 1 study has recently shown a long-time stabilization and partial response in patients with metastatic non-small cell lung cancer (NSCLC) [145].

COX2 induces the production of prostaglandin as PGE2, which stimulates IDO activity [146]. Celecoxib, a COX2 inhibitor in association with adoptive therapy, enhances the antitumor effect of the vaccinotherapy with an increased survival rate, linked to the increased cytotoxicity of T cells and the decreased amounts of tumor-associated IDO [147]. Another model of lung cancer showed that COX2 inhibitors decrease IDO and Foxp3 expression, meaning that these treatments can inhibit Treg differentiation [148].

Ethyl pyruvate, an inhibitor of NF-κB, can also potently inhibit IDO expression [149]. Imatinib, used in gastrointestinal stromal tumor (GIST) or chronic myeloid leukemia (CML), can also inhibit IDO expression, by inducing Treg apoptosis and activation of CD8^+^ T cells [150]. In their mouse model of GIST, they showed reduced tumor growth with imatinib and reduced IDO expression. This effect was reduced in the case of treatment with kynurenine catabolites. Imatinib seems to reverse the Warburg effect in leukemia models, diminishing glucose uptake [151]. Finally, the IDO1 silencing could also be a promising option for treating IDO-expressing cancers [152]. Indeed, in vitro and in vivo studies have shown that IDO1 silencing can suppress tumor growth and restore T cell immunity in melanoma models. In mouse models, decreased gene expression of IDO1 by shRNAs leads to the inhibition of tumor growth [153].

### 3.3. Isocitrate Dehydrogenase (IDH)

#### 3.3.1. Biochemistry of IDH

IDH has three isoforms IDH1, IDH2 and IDH3, encoded by five genes (IDH1, IDH2, IDH3A, IDH3B and IDH3G) [154]. IDH1 is expressed in the cytosol of eukaryotic cells and also in peroxisomes, whereas IDH2 and IDH3 are expressed in the mitochondria. IDH1 catalyzes the conversion of isocitrate from glucose metabolism into α-ketoglutarate (α-KG) via oxidative decarboxylation. Glutamine can also provide α-KG for IDH enzymes. It permits the reduction of NADP^+^ into NADPH and the production of CO_2_. This reaction is reversible, restoring the isocitrate that can be further metabolized into acetyl-coA. Acetyl-CoA participates in lipid biosynthesis and protein acetylation [155,156]. The product of IDH1 activity is an intermediate of the TCA under normoxic conditions, whereas IDH1 catalyzes the reverse reaction (converting α-KG into isocitrate) under hypoxic conditions.

#### 3.3.2. IDH Expression in Tumor Cells

IDH1 and IDH2 have different expression patterns in tumors [157]. IDH1 mutations were first described in central tumors and acute myeloid leukemia (AML), and then in the number of hematopoietic neoplasms (myelodysplastic syndrome, T cell lymphoma) and sarcomas (chondrosarcoma, central chordoma, periosteal chondroma) [158]. The IDH1 gene has numerous missense mutations leading to the substitution of an arginine at codon site 132 of the catalytic pocket that binds isocitrate and NADPH. The most frequent is its substitution for a histidine (R132H), and mutations of IDH2 occur most frequently at arginine 172 or arginine 140 [159]. The IDH1 mutant is found in above 80% of grade II-III glioma tumors and secondary glioblastoma [160]. This mutation seems to occur early in gliomagenesis, prior to other genomic alterations [161]. Its expression is different between primary and secondary glioblastoma. Indeed, primary glioblastomas (GBMs) are associated with PTEN loss, EGFR amplification and chromosome 10 loss of heterozygosity, whereas secondary ones are associated with ATRX mutations, loss of p53 and chromosome 19 LOH [158]. It has been shown that IDH1 mutations are almost systematically associated with either TP53 mutation or 1p19q co-deletion [162]. Furthermore, other genomic abnormalities have been identified: HoxA9, Flt3, ATRX, TERT and NRas [159]. IDH1 is not sufficient to induce tumors alone and needs other genomic alterations. IDH cooperates with these alterations in order to induce cancer.

**Figure 4:**
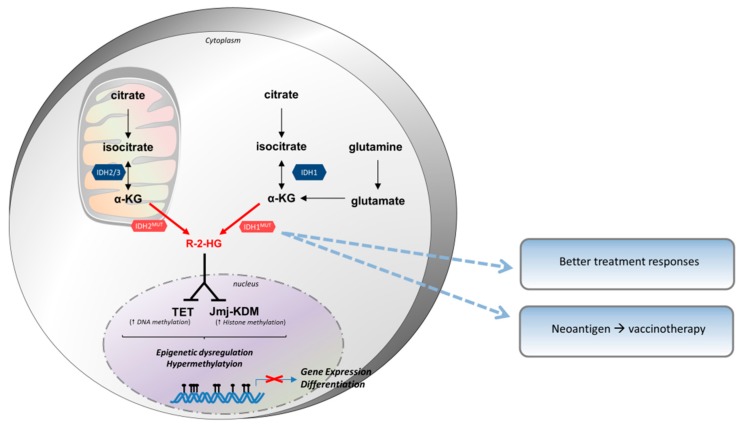
Isocitrate dehydrogenase (IDH) mutations in cancer cells and their clinical impact. Wild-type IDH1 (cytosol) and IDH2/3 (mitochondria) catabolize the oxidative decarboxylation of isocitrate into α-ketoglutarate (α-KG). This reaction generates CO_2_ and replenishes NADPH pools in the cell. IDH mutants (IDH^MUT^) display a reduced affinity for their natural substrate, while gaining neomorphic activity to convert α-KG into R-2-hydroxyglutarate (R-2-HG). R-2-HG is an oncometabolite that plays a key role in tumor progression. It is usually kept at low levels as it is re-converted into α-KG by dehydrogenases. In IDH mutated cancer cells, these dehydrogenases are overwhelmed by mutant activity, generating very high levels of R-2-HG. R-2-HG principally acts by binding to 2-OG-dependent dioxygenases, such as TET (TET methylcytosine dioxygenase) and Jmj-KDM (Jumonji C domain histone lysine demethylase) proteins. The binding to TET and KDM inhibits activity, increasing DNA and histone methylation patterns, respectively, and, thereby, altering gene expression patterns and cell differentiation. Patients with IDH^MUT^ tumors seem to respond better to treatment. Moreover, this mutation leads to the expression of a neoantigen that can be a target for vaccinotherapy.

The affinity of mutant IDH1 to isocitrate decreases but its affinity for α-KG increases, favoring the neomorphic activity compared to normal activity [163]. The IDH1 mutant enzyme catalyzes the transformation of α-KG into R-2-hydroxyglutarate (R-2-HG), consuming NADPH [164]. R-2-HG is an oncometabolite and is necessary to maintain tumor phenotype in IDH1 mutant cells (Figure 4). It is maintained at low concentrations by the 2-HG dehydrogenase activity in normal cells which recycles 2-HG into α-KG. Mutant IDH1 produces high concentrations of 2-HG, overtaking its degradation or recycling [164]. Its accumulation in the brain leads to a higher risk of tumor development. Indeed, patients with 2-HG dehydrogenase constitutive deficit have a higher rate of brain tumors [165].

Furthermore, the mutation that leads to the oncogenic activation of the enzyme, also alters the interaction between isocitrate and IDH1 [166]. In vitro, this enzyme has an 80% reduction of its catalytic activity and an alteration of its affinity for its substrate [167]. By forming heterodimers with a wild-type form of IDH1, it dominantly inhibits the normal enzyme, leading to an impairment of IDH1 activity compared with wild-type homodimer and to a reduced production of α-KG. Formation of R-2-HG requires heterozygosity of the IDH1 locus, whereas homozygous IDH1 mutations have shown reduced levels of R-2-HG [168]. Indeed, the IDH1 mutant converts α-KG into R-2-HG only if IDH1 wild-type produces α-KG, permitting higher R-2-HG production. Reduction of NADPH production resulting from an IDH1 mutation could also contribute to tumorigenesis. Indeed, the expression of mutant IDH1 in mouse models has a pro-leukemic effect, by leading to an impairment of hematopoietic differentiation and an increased expression of stem cell markers [169]. IDH1 mutation is associated with increased oxidative stress with elevated ROS levels, contributing to an increased risk of cancer [170].

R-2-HG is an “oncometabolite” in glioma cells and AML, and other solid tumors [171]. It is a competitive inhibitor of 2-OG-dependent dioxygenase, by binding to the α-KG binding site in the catalytic pocket. Indeed, it blocks these enzymes according to α-KG bioavailability. 2-OG-dependent dioxygenase are a family of enzymes involved in the modification of the methylation of DNA and histones. This leads to a hypermethylated state through the inhibition of TET proteins which normally remove DNA methylation [163], and of the Jumanji family which removes histone methylation. IDH seems to be involved in several steps of tumorigenesis. Indeed, mouse models treated with IDH2 inhibitors had decreased R-2-HG production, of the tumor growth, but not their differentiation [172]. High levels of R-2-HG also inhibit PHD (prolyl hydroxylases) and the degradation of HIF1-α, favoring tumor formation, growth and survival through the production of vascular endothelial growth factor (VEGF), glucose transporter 1 (GLUT1), phosphoglycerate kinase 1 (PGK1) [158]. A study showed that transfection of glioma cells with IDH1 mutation leads to the upregulation of HIF1-α and to increased tumor cell proliferation [173]. The IDH1 mutant also seems to induce the NF-κB pathway in an HIF1-α dependent manner, regulating tumor cell proliferation. Together, these data mean that tumor cells can block cellular differentiation through metabolite-induced epigenetic modifications [174,175], but other mutations are probably needed for their progression and survival.

#### 3.3.3. Impact in Clinical Routine

Patients harboring an IDH1-mutated tumor seem to present better treatment responses than non-IDH1 patients [176]. Opposite of other molecules discussed above, IDH1 do not inhibit the function of immune cells, but are immunogenic. The IDH1 mutation is associated with better survival in glioblastoma patients (3.8 years) versus wild-type IDH1 (1.1 years) [177]. IDH1 (R132H) encodes a neoantigen widely and prematurely expressed by all tumor cells. Furthermore, it is recognized by CD4^+^ T cells which, once activated, produce IFN-γ. Indeed, IDH1 contains an immunogenic epitope that can be a target for vaccinotherapy. Peptides are presented by the MHC II machinery and induce activation of CD4^+^ specific T cells with a Th1 polarization and humoral response [178]. There is also probably a CD8^+^ immune response [179]. In mouse models, vaccination led to an efficient antitumor immune response, controlling tumor proliferation. This effect was not present when vaccinated with wild-type IDH1, meaning that vaccinotherapy could have low side effects. There was a significantly lower expression of immunosuppressive components including VEGF, HIF-1α, and IL-10, and increased expression of IFN-γ, granzyme B and perforin. Like with other therapeutic strategies, tumors have developed a resistance to vaccinotherapy by decreasing the expression of the neoantigen. Tumors which expressed IDH1 but resisted vaccinotherapy exhibited reduced IDH1 (R132H) expression. Two clinical trials are actively recruiting grade II glioma (NCT02193347) and grade III/IV glioblastoma (NCT02454634) patients, assessing safety and tolerability of anti-IDH1(R132H) peptide vaccine.

### 3.4. Hypoxic Conditions

#### 3.4.1. Characteristics of Hypoxia Conditions

Hypoxia is a common feature of tumor tissues. It arises because of oxygen diffusion limitations and an abnormal vasculature. Hypoxia has many effects on tumor phenotypes, mostly driven by the selection of hypoxia-dependent genes such as HIF-1α. HIF-1α is a master regulator of oxygen homeostasis. It is the central molecule mediating the response to hypoxia conditions, permitting the adaptation of cell transcriptional programs. HIF-1 is a heterodimer of two subunits: HIF-1α and HIF-1β [180]. Both subunits interact with DNA, but the α-subunit cross-links more strongly. HIF-1α is produced in response to a decrease in cellular O_2_ concentrations in an exponential manner [181], whereas HIF-1β is constitutively expressed. HIF-1α has a post-translational regulation, dependent on oxygen availability, via the hydroxylation of its proline and asparagine residues [182,183].

Under well-oxygenated conditions, it is hydroxylated by the prolyl hydroxylase 2 (PHD2), active only in the presence of O_2_. Its hydroxylation permits the binding of the von Hippel-Lindau (VHL) protein. VHL is a tumor suppressor protein, because its interaction with HIF-1α leads to the recruitment of an E3 ubiquitin ligase, targeting HIF-1α to the proteasome and its degradation [184]. PHD2 uses one oxygen atom from O_2_ for its catalytic activity, and the other is inserted in α-KG, producing CO_2_ and succinate [185]. Under hypoxia, PHD2 is inactive, HIF-1α becomes stable, and it interacts with co-activators such as p300/CREB-binding protein (CBP) through its transactivation domain. HIF-1α can translocate to the nucleus and bind to HIF-1β. The heterodimer can then regulate the transcription of several genes. The hydroxylation of asparagine residues of the HIF protein through factor inhibiting HIF-1 (FIH-1) activation inhibits the binding of p300/CBP co-activator [186]. FIH-deficient mice have increased HIF-1 activity, and FIH-1 seems to also have a metabolic role by decreasing ATP cellular levels and by the induction of 5’AMP-activated protein kinase (AMPK), leading to a hypometabolic state [187].

HIF-1α is a transcription factor of genes containing, in their promoters, hypoxia response consensus sequences [188]. Indeed, these genes are involved in decreased O_2_ consumption and/or increased O_2_ delivery. One of the most important genes is VEGF, which is involved in neoangiogenesis. Furthermore, LDH-A and pyruvate dehydrogenase kinase 1 (PDK1) are also targets of HIF-1α, permitting the switch from OXPHOS to aerobic glycolysis [189,190].

#### 3.4.2. Hypoxia and Tumor Cells

Tumors commonly overexpress HIF-1α, and this phenomenon is due to several mechanisms. The main mechanism is the micro-environmental hypoxia conditions, because of high rate of cell proliferation, and impaired angiogenesis with low oxygen rates at the center of the tumor regions. HIF-1α expression can also be regulated in an oxygen-independent manner. Indeed, growth signaling pathway alterations, such as Pi3K/Akt/mTOR or MAPK, can favor activation and translation [191,192]. The tumor suppressor gene p53 is also involved. Indeed, p53 promotes the ubiquitination of HIF-1α in a mouse double minute 2 homolog (Mdm2)-dependent manner and its loss led to increased HIF levels and increased VEGF production in the in vivo models [193]. Others tumor suppressors, such as the loss of function of FH (fumarate hydratase), SDH (succinate dehydrogenase) or VLH can lead to a pro-tumorigenic role [194]. Some oncometabolites such as fumarate and succinate, can also induce HIF stability through the inhibition of PHD2 [185].

HIF proteins induce the expression of several genes involved in tumor phenotypes, such as platelet-derived growth factor (PDGF), transforming growth factor alpha and beta (TGF-α, TGF-β), matrix metallopeptidase 1 (MMP1), C-X-C chemokine receptor type 4 (CXCR4) [195]. Activated HIF-1α has a crucial role in the adaptive response of tumor cells to oxygen conditions in their microenvironment. HIF-1α overexpression has several effects on tumor cell phenotypes: immortalization, stem cell maintenance, epithelial to mesenchymal transition, protumoral autocrine signaling, metabolic reprogramming, invasion, radioresistance and neoangiogenesis [196]. As discussed above, hypoxic tumor cells tend to shift their metabolism from OXPHOS to aerobic glycolysis in order to maintain their energy production. HIF-1α induces enzymes of the glycolytic pathway (LDH-A, PKM, hexokinases) and the overexpression of GLUT1 transporters, in order to increase glucose uptake. Another important effect is the regulation of neoangiogenesis through VEGF and nitric oxide species (NOS) production [197]. HIF-1α also promotes metastasis through the upregulation of oncogenes such as TGF-β and EGF. Finally, HIF expression participates in immune escape. Indeed, hypoxia induces the autophagy of tumor cells [198], thereby resisting the cytolytic activity of immune cells.

#### 3.4.3. Effect of Hypoxia on Immune Cell Types

**Figure 5:**
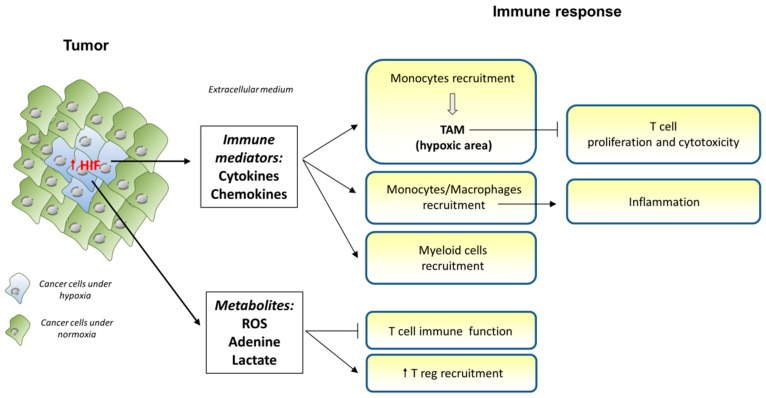
Consequences of hypoxia and HIF-1 production by cancer cells on the immune response. While proliferating, cancer cells develop hypoxic regions at the center of the tumor. This results in HIF-1α stabilization and expression. HIF-1α triggers the expression and release of several cytokines and chemokines that attract monocytes, macrophages and myeloid cells into these regions. In some cancers, monocytes differentiate into TAMs (tumor-associated macrophages) that impair T cell proliferation and cytotoxic properties. In other cases, recruited macrophages trigger inflammation, promoting cancer progression. Some myeloid cells like MDSCs contribute to immunosuppression. Finally, O_2_-deprived cancer cells may also produce and release metabolites such as ROS, adenine and lactate that will further block T cell function and increase the recruitment of Tregs with immunosuppressive functions.

Tumor-associated macrophages (TAMs) have an HIF-dependent pro-tumoral effect and the hypoxic areas of tumors are enriched in TAMs due to the secretion of several chemokines from tumor cells (Figure 5). Indeed, the deletion of HIF-1α in TAMs led to reduced tumor progression in the mouse model of breast cancer [199]. This effect was independent of VEGF or reduced vascularization, meaning that there is another mechanism in place. In this mouse model, it was shown that TAMs inhibit T cell proliferation and cytotoxicity under hypoxic conditions.

HIF-1α and HIF-2α have different roles in angiogenesis. Indeed, in a murine model of melanoma, it has been shown that HIF-2α is involved in the production of soluble vascular endothelial growth factor receptor (VEGFR) which neutralizes VEGF, with antitumor effects [200], whereas HIF-1α induces VEGF production and neoangiogenesis. Indeed, HIF-1α induces the secretion of stromal cell-derived factor-1 alpha (SDF-1α) by tumor cells in the glioblastoma model, which is a chemokine that potently attracts monocytes/macrophages to promote inflammation, angiogenesis and invasion [201]. Furthermore, HIF-1α induces the recruitment of endothelial and pericyte progenitor cells to promote angiogenesis in this model.

Hypoxia contributes to the recruitment of myeloid cells from bone marrow. Indeed, HIF-1α induces the production of chemokines and the expression of their receptors [202], including CCL5 or CXCL12 [201,203]. Furthermore, HIF-1α induces the expression and secretion of soluble molecules such as VEGF, which, besides their proangiogenic role, have a chemoattractant function for myeloid cells [204], or endothelins by tumor cells [205].

Hypoxia in tumor cells can suppress T cell immune function through the production of three metabolites: ROS, adenosine and lactate. Hypoxia inhibits adenosine deaminase and adenosine kinase, increases the catalytic activity of nucleotidases CD39 and CD73, and inhibits the expression of transporters involved in adenosine uptake [206,207]. Hypoxia also inhibits T cells through the production of oxygen free radicals and the HIF-1α control of several immunosuppressive genes. ROS are produced by tumor cells and stromal cells and are released in the extracellular medium because of the less efficient respiratory chain in mitochondria [208]. They inhibit NF-κB translocation to the nucleus and inhibit cytokine production. Lastly, ROS partly induce the degradation of the TCR complex including CD3ζ [209]. Hypoxic tumor cells enhance Treg recruitment, through the production of CCL28 which interacts with CXCR10 (a proangiogenic and immunosuppressive molecule) [210], the expression of neuropilin-1 (NP-1) which attracts Tregs in response to VEGF [211], or the secretion of the chemoattractant TGF-β by tumor cells [212]. Finally, HIF-1α contributes to the aerobic glycolysis in cancer cells by upregulating several key glycolytic enzymes, thereby increasing the uptake of key nutrients including glucose. Importantly, this increase of glucose uptake by cancer cells has been shown to deprive effector T cells of nutrients, thereby impairing their antitumor immune function [26]. Interestingly, the same group has demonstrated that the high glycolytic rate in cancer cells upregulates the expression of the key T cell co-inhibitor ligand PDL-1 [26]. It remains to determine if the effects of cancer metabolism can be extended to the regulation of other T cell co-stimulatory molecules, and potentially map out which underlying particular metabolic pathways are critical to this process.

#### 3.4.4. Targeting Hypoxia

Several molecules can inhibit HIF-1α and hypoxia at different levels [213]: mRNA expression and translation, protein stability, HIF dimerization, its binding to DNA on its specific response elements, or inhibition of its transcriptional activity.

The inhibitors of mRNA expression are important to note. mRNA expressions can be blocked by using an antisense oligonucleotide, EZN-2968 [214]. This molecule has shown an efficient reduction of mRNA levels and tumor progression in vitro and in vivo [183], and a Phase 1b is actually ongoing in hepatocellular carcinoma (HCC) (NCT02564614). Aminoflavone is another HIF-1α inhibitor at mRNA level. It normally binds to AhR [215]. In vitro, aminoflavone inhibits HIF-1α in an AhR-independent manner. Studies have been conducted to assess its safety and tolerability and results are expected. There are no ongoing clinical trials assessing this molecule. Others molecules have shown antitumor effects. PX-478 is a melphalan derivative that suppresses constitutive and hypoxia-induced expression of HIF-1α. Thus, the association of gemcitabine and PX-478 have shown significant inhibition of tumor growth and favors gemcitabine-induced immune response [216].

Several molecules can decrease HIF-1α biosynthesis, among which are inhibitors of topoisomerase, receptor tyrosine kinase, cyclin-dependent kinase (CDK), activators of p53 and microtubule disrupting agents [213]. Topotecan is the most used molecule which blocks protein translation. It is a camptothecin analogue that inhibits topoisomerase I by stabilizing Top1-DNA cleavage complexes. This leads to double strand DNA breaks, inducing cell apoptosis. This effect is not dependent on DNA damages [217]. Thus, xenograft models have shown that chronic administration of topotecan inhibits HIF-1α expression, angiogenesis and tumor proliferation [218]. A study has been completed assessing the effect of oral daily topotecan on the inhibition of angiogenesis in refractory neoplasms (NCT00117013). Cardiac glycosides are also molecules that can inhibit HIF-1α, of which digoxin is one [219]. In the in vivo models, digoxin has shown antitumor effects, with decreased xenograft growth. Enforced expression of HIF-1α through transfection induces a resistance to antitumor immune response. Two clinical trials are ongoing to assess digoxin antitumor effects (NCT02212639 and NCT01763931). Inhibition of the mTOR pathway can also be used to inhibit HIF-1α translation. They inhibit HIF transcription at different levels. Several molecules are currently being developed in preclinical models in order to inhibit HIF-1α immunosuppression. These molecules act at different levels, such as protein dimerization (acriflavin), its binding to DNA (echinomycin or anthracyclines), or its transcriptional activity (bortezomib or chemotin). There is no ongoing clinical trial to assess the therapeutic effects in tumor patients.

## 4. Conclusions

The past years have shed light on the tremendous impact of tumor metabolic reprogramming on the immune response. As immune escape is one of the main features of tumor progression, hampering the body’s control over its own abnormal cells, it has paved the way to novel therapeutic avenues with incredible responses (as long as patients can respond). The influence of cancer cell metabolic switch on this phenomenon provides new therapeutic strategies in the clinic to reeducate the immune system and eradicate tumor cells. Even though cancer cells use multiple metabolic methods to subvert immune cells from their fundamental role (lactate; IDO, HIF1-α, and probably others), targeting these metabolic pathways together with immune checkpoint inhibitors might, in some settings, improve the clinical response. As discussed throughout this review, it is now evident that soluble metabolites released from cancer cells control the fate of immune cells by modifying and altering the composition of the environmental milieu. It will be of interest to investigate whether membrane bound molecules expressed by cancer cells, especially TCR ligands including neoantigens or co-stimulatory molecules involved in priming/activating the antitumor immune system may also be affected by such metabolic rewiring.
